# Information Management for the Assistive Technology Provision in Community: Perspectives of Local Policymakers and Health Service Providers

**DOI:** 10.1155/2018/8019283

**Published:** 2018-02-28

**Authors:** Suchitporn Lersilp, Supawadee Putthinoi, Sayaka Okahashi

**Affiliations:** ^1^Department of Occupational Therapy, Faculty of Associated Medical Sciences, Chiang Mai University, Chiang Mai, Thailand; ^2^Division of Occupational Therapy, Department of Human Health Sciences, Graduate School of Medicine, Kyoto University, Kyoto, Japan

## Abstract

**Background:**

Assistive technology (AT) is a way to enhance the performance of daily activities in people with disabilities and help them live more independently. However, an important problem in providing AT lies in the effectiveness of information management. Local policymakers and health service providers have become aware of this problem and their perspectives are the key to solving it.

**Methods:**

This study explored the types of AT provided for people with disabilities in the community and the perspectives on information management of local policymakers and health service providers. A survey checklist and semistructured in-depth interview were the instruments of this study. The key informants in this study included administrators, nurses, and physical therapists from four community areas in Chiang Mai, Thailand.

**Results:**

The medical records showed that the types of AT provided were mostly second-hand (57.24%) and borrowed devices (57.73%). All of them were low-tech devices (crutch, cane, walker, wheelchair, and adaptive tricycle). In addition, the results indicated three perspective aspects related to information management: (1) problems in the database recording system, (2) different policies and processes of information management, and (3) improvement of the AT provision system.

**Conclusion:**

The perspectives of local policymakers and health service providers indicated related problems, impacts of policies, and ways to improve the AT provision system by applying information technology.

## 1. Introduction

Information management is essential for a successful community and district health system, where the information from local, national, and other available resources is linked. District health systems for primary health care are concerned with health systems management and other sectors such as health care service providers, district health managers, and agencies [1]. Willem and Buelens [2] suggested that information from other units can provide interunit knowledge sharing to enhance a positive organizational outcome. Management of information from one or more sources in community health at the district health level is useful for both community and national policy. Information in the community helps the health service providers and public health officials direct the community in meeting its needs. Village health volunteers (VHVs) in Thailand are key persons in gathering data on community resources for national policy, and community-based resource management requires information from many different related groups such as community hospitals, local governments, and village health volunteers. The information from each group is used for support in qualifying disability rights and making decisions in providing direct and indirect services and disability benefits. Jokandan et al. [3] showed that Information and Communication Technology (ICT) had heavy impact on the formality of municipal and urban management as well as all employees. ICT functions are able to manage the complex information of community health services and provide assistive technology (AT) in community contexts. Thus, this study aimed to explore the information process among the people at the community level.

Assistive technology (AT) includes adaptive and assistive devices that enable improved performance in living independently for people with disabilities. AT not only impacts these people directly, but also could provide caregivers with immediate relief, reduced stress, and help in providing care more easily and safely [4]. Mann et al. [5] found that AT and home modifications can reduce home care costs.

Many countries have laws and legislations regarding AT: for example, Bausch and Hasselbring [6] reported that types of AT and related services were regulated many years ago in the Individuals with Disabilities Act Amendments PL 105-17 (USA). However, many studies reported that people with disabilities could not use the technologies because of the time it takes to learn, train in, and practice their use, and some clients refused to use them. Besides the problem of use, the provision of AT is an important issue for people with disabilities in the community. Indeed, some communities receive a great deal of AT from the government and through donations, while many communities lack some types of the technology. Different situations create various problems; for example, some communities have maintenance and storage problems, while others lack AT. A main cause of this situation is the need for an information management system or an incomplete one. Based on the concept of “access for all,” AT could make an important contribution to the care of dependent people, such as those with disabilities, children with special needs, and elderly people in institutions and at home. This could be carried out by video-monitoring, remote health monitoring, electronic sensors, assistive devices, and adaptive equipment [7]. Cooper et al. [8] pointed out that not only modern or high technologies, but also problem trends in developing AT are related to ineffective delivery systems, changes to public policy, and coordination among people with disabilities, policymakers, manufacturers, researchers, and service providers. These concerns highlight the current ineffectiveness of AT. Ocepek et al. [9] reported that using AT improved quality of life in the majority of the participants. AT was recommended to the user mostly by a general practitioner or consultant physician, nurse, specialist, or relatives, as there are no AT specialists in community hospitals or community healthcare centers. As medical teams or a public health team carry out work in the municipality, the information management system providing AT is recorded in medical or informal records that are difficult to locate. For these reasons, this study explored the types of AT and perspectives of local policymakers and health service providers on information management in providing AT for people with disabilities in the community.

## 2. Materials and Methods

This study used quantitative and qualitative research methods in four community areas in Chiang Mai, Thailand. A survey checklist explored the types of AT provided for people with disability in the community, and a semistructured in-depth interview was used to study the perspectives of local policymakers and health service providers.

**Figure 1:**
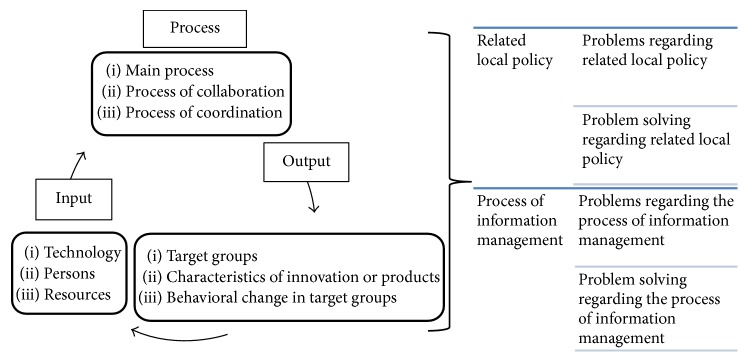
The information management system in community health at the district health level.

A survey method was planned and processed for the objective of exploring types of AT provided for people with disabilities in the community. Information was collected during 2015 and 2016 from secondary sources of data in the medical and informal records of community hospitals and subdistrict municipalities in four community areas of Chiang Mai province, Thailand. In addition, a semistructured in-depth interview was administered for the objective of studying the perspectives of local policymakers and health service providers on information management in providing AT for people with disabilities in the community. This objective applied to the information management system in community health at the district health level [10] in order to create the scope of the interview, as shown in Figure 1.

Probing questions comprised four points as follows:

1. Related local policy of AT provision for people with disabilities in the community
2. Problems and problem solving regarding related local policy of AT provision for people with disabilities in the community
3. Process of information management regarding AT provision for people with disabilities in the community
4. Problems and problem solving regarding the process of information management regarding AT provision for people with disabilities in the community

There were 16 key informants in this study comprising 10 administrators of subdistrict municipalities and 6 health service providers, including nurses, physical therapists, and community-developers of local community hospitals in four community areas in Chiang Mai, Thailand. They were interviewed and recorded individually. Data from interviewing were analyzed by content analysis.

## 3. Results and Discussion

The results showed that all four community areas did not have a database management system of AT provision, which was recorded in the hospitals' general medical records for 2015 and 2016. It was found that 289 people with disability were provided with AT in the four community areas. The results showed that 55.71% and 44.29% were female and male, respectively. Besides gender, there was no other personal information in the database of these people. In addition, the types of AT provided were mostly second-hand (57.24%) and borrowed devices (57.59%). One person was provided with two AT devices, a walker and cane; therefore, the number of AT devices was greater than that of people with disabilities. All of the devices were low-tech such as a crutch, cane, walker, wheelchair, and an adaptive tricycle, as shown in Table 1.

**Figure 2:**
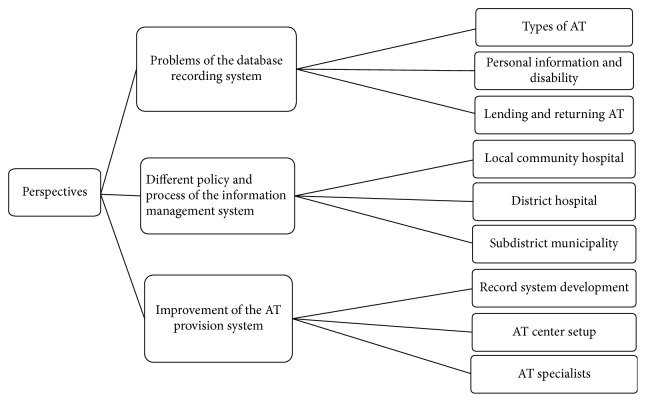
Perspectives of local policymakers and health service providers on information management in providing AT in the community.

In addition, the results indicated three perspective aspects related to the information management system that emerged from the local policymakers and health service providers. The first aspect was a problem in database records, including lack of a type of AT system, lack of personal and disability information of clients receiving AT, and an incomplete system of lending and returning AT. The second aspect was the policy and process of different organizations. There are three main related organizations providing AT; the local community hospital, district hospital, and subdistrict municipality. Policies of the local community and district hospital come under the Provincial Health Office. On the other hand, those of the subdistrict municipality come from the mayor. Therefore, there are different policies and processes of information management regarding AT provision. The local community hospital has a system of finding people in the community who need AT, assessing which AT meets their needs, coordinating with district hospitals for receiving AT, and providing AT directly to the people, as well as participating in home visit teams with the district hospital and subdistrict municipality. District hospitals that participate in home visit teams with the subdistrict municipality directly provide evaluation, training, and repair of AT (both new and used) and issue certificates of disabilities. Subdistrict municipalities register people with disabilities in the community in order to provide living allowance, home visits, and coordination with the provincial hospital or disability center that receives AT. The last aspect was improvement of the AT provision system. The local policymakers and health service providers pointed out the importance of a lending system, which is a way to manage and exchange second-hand AT between communities. Furthermore, they indicated that when the lending system is linked between communities, the information management system and resource sharing occur, as well as an increase in the efficiency of services for the people in the community. In addition, they also thought of setting up the AT center in the community, as a hub of sharing information, demonstrating, evaluating, training and repairing AT, and modifying the home for independent living. Moreover, the AT specialists provide services that help to decrease waiting times and transportation or traveling costs (as it is extremely difficult for some clients to get to and from the hospital). Content analysis of these perspectives is shown in Figure 2.

The findings indicated that the types of AT provided in the community were low-tech and used for the purpose of mobility. These findings were related to Putthinoi et al. [11] in that AT homes of the elderly were assistive products and technology for personal use in daily living, indoor-outdoor mobility, and transportation. This was because the legal rights of people with disabilities in Thailand are still limited by government budgets. Low-tech AT devices are inexpensive, simple to make, and easily obtainable for all [12]. This result relates to Lersilp [13], who indicated that although there was a related regulation and an assistive technology provision system in each university, many types of assistive technologies were not used, and some were not even received.

Furthermore, these findings were fundamental to the design of an effective information management system by applying information technology in providing AT for people with disabilities in the community. According to the above results, it was found that the database records on providing AT in community hospitals and subdistrict municipalities were recorded in informal notebooks and did not contain any personal information, except for name and gender. This was because the community hospitals and subdistrict municipalities were not responsible for providing AT directly. Community hospitals have the role of a hybrid health service, including health promotion, disease prevention and control, medical treatment, rehabilitation, and health risk factor management [14]. AT provision is a part of rehabilitation; therefore, no AT specialist or specific AT officer is responsible for the database recording system in community hospitals.

The results above show that communities incur problems of the database recording system and related policy. According to Lersilp et al. [15], this information shows that a major problem in the provision and use of AT was the criteria of educational policy. When developed countries are considered in similar contexts, Japan is a good model of AT provision, as the rental system is an interesting AT provision system in that country. After hospital discharge, people or local residents usually start using AT rental services under Long-Term Care Insurance, which came into force in 2000. Approximately 67% of these people receive advice from a care manager and about 68% select AT rental agencies near their home through the “care manager advisory,” usually without comparing prices. People pay only 10% of the rental charge under Long-Term Care Insurance. Although prices are not the same between agencies, they are often within the standard price area [16].

The charge in this system can be determined freely for some products by each AT rental agency. Wholesale AT rental agencies are responsible for profit and general, distribution, and maintenance costs; consecutively, while “in-house” or retail rental agencies are responsible for maintenance cost, profit, and general and distribution costs, consecutively. Sometimes people buy their own AT according to specific needs, while considering the rental charge per month, purchase price, frequency of everyday use, and so on. Unfortunately, there are no good websites such as “comparison shopping websites” providing comprehensive information that shows comparisons of specific AT rental charges or purchases prices [17, 18].

Care managers, who are under Long-Term Care Insurance, play an important role in managing each person's future plan of care/rehabilitation service and AT rental according to the severity of disability. However, it is possible that they only have information on AT depending on their own specialty (e.g., nurse and dentist). Important information includes not only the rental details, but also AT safety, especially physical safety after long-term use. Some cases indicated the need for AT that had been discontinued. The AT business model should be considered in terms of the AT distribution system. Manufacturers of AT mainly contact rental agencies without having information of individual users, thus making it difficult for the former to understand user needs [16–18]. An AT supply system set up by the medical and welfare combination team will be needed to solve these problems.

Regarding Japanese Occupational therapists, there are 47 branches of the Japan Association of Occupational Therapists in all prefectures of Japan. Thirty-four of them have had the “AT advice system” since July 2014 to support occupational therapists who have problems with AT matching/adaptation for their patients. Several occupational therapists, who have specialized in AT, are registered as mentors in each branch, and inexperienced occupational therapists can communicate with them whenever necessary. It is important to set up both a hard and soft AT supply system in the aging community [17, 18].

## 4. Conclusion

This study explored the types of AT provided for people with disabilities in the community and the perspectives on information management of local policymakers and health service providers. Results showed that 289 people with disabilities were provided with AT in the study areas. The types of AT provided were mostly second-hand and borrowed devices. All of them were low-tech such as a crutch, cane, walker, wheelchair, and an adaptive tricycle. However, it was found that the hospitals' general medical records of information on AT provision were incomplete in the AT database and contained no personal information of people receiving AT. The local policymakers and health service providers reflected three perspective aspects related to the information management system such as problems of the database recording system, different policies and processes of the information management system, and improvement of the AT provision system. These findings were fundamental to the development of an effective information management system providing AT for people with disabilities in the community by applying information technology.

## Figures and Tables

**Table 1:** AT provision information collected from community hospitals (2015, 2016).

Information	Number	Percentage
*Type of AT*		
Crutch	80	27.59
Cane	80	27.59
Walker	69	23.79
Wheelchair	57	19.66
Adaptive tricycle	4	1.38
*Total*	*290*	*100.00*
*Category of AT*		
Second-hand device	166	57.24
New device	124	42.76
*Total*	*290*	*100.00*
*Way of provision*		
Borrowed	167	57.59
Disability benefits	123	42.41
*Total*	*290*	*100.00*
